# Overview of systematic reviews on Chinese patented oral medicines for promoting blood circulation and removing blood stasis combined with western medicine in the treatment of coronary heart disease angina pectoris

**DOI:** 10.3389/fcvm.2025.1553735

**Published:** 2025-06-20

**Authors:** Xinyi Chen, Huan Liu, Hongli Wu, Yi Deng, Wenxi Peng, Yanming Xie, Junjie Jiang

**Affiliations:** Institute of Basic Research in Clinical Medicine, China Academy of Chinese Medical Sciences, Beijing, China

**Keywords:** Chinese patented oral medicines, coronary heart disease, angina pectoris, systematic review, overview, meta-analysis

## Abstract

**Objective:**

To evaluate the methodology quality, reporting quality, and evidence quality of systematic reviews and meta-analyses on Chinese patented oral medicines that promote blood circulation and remove blood stasis combined with conventional Western medicine in the treatment of coronary heart disease angina pectoris. The aim is to identify and address methodological issues in systematic reviews of Chinese patented oral medicines for promoting blood circulation and removing blood stasis in angina pectoris. This study also offers methodological guidance for future research design and implementation, and provides a basis for clinical decision-making.

**Methods:**

A systematic search was performed using CNKI, Wanfang, VIP, CBM, PubMed, Cochrane Library, Embase, and Web of Science databases, covering the period from the inception of each database to July 18, 2024, and meta-analyses on randomized controlled trials were included. Methodological quality was evaluated using AMSTAR-2, reporting quality using PRISMA 2020, and evidence quality of the outcome indicators using GRADE.

**Results:**

Twenty meta-analyses were examined, involving a total of 41,231 patients with angina pectoris. The methodological quality of all studies was rated as “critically low,” with notable deficiencies in the registration of the study protocol, study inclusion criteria, assessment of individual study risk of bias, evaluation of the likelihood of publication bias, and discussion of the effects of publication bias on the results. The assessment of the three qualities mentioned above revealed common issues, including incomplete abstracts, lack of characteristics for pooled outcomes, failure to report risk of bias across studies, missing registration information, lack of accessibility details for protocols, and unreported modifications to registered protocols or plans. Evidence quality assessment revealed that 16 outcome indicators were rated as “moderate,” 29 as “low,” and 46 as “very low.”

**Conclusion:**

Despite the demonstrated efficacy and safety of Chinese patented oral medicines that promote blood circulation and remove blood stasis in the adjuvant treatment of angina pectoris, the low methodological and reporting quality of current systematic reviews and meta-analyses compromises the reliability of these findings. Future research should focus on standardizing study design and reporting to improve the reliability of evidence.

## 1 Introduction

Coronary heart disease (CHD) is characterized by the deposition of lipids and proliferation of fibrous tissue in the walls of coronary arteries, resulting in the formation of plaques that leads to vascular narrowing or occlusion. This process causes myocardial ischemia, hypoxia, and, in severe cases, necrosis (1). Globally, CHD represents the leading causes of death (2). In the United States, approximately 360,000 deaths were attributed to CHD in 2019 (3). In China, cardiovascular disease (CVD) caused a total of 4,584,273 deaths in 2019, accounting for 22.26% of global CVD-related deaths. CVD mortality in rural and urban areas accounted for 46.74% and 44.26% of all deaths, respectively. CVD prevalence in China is due to the rapid increase in atherosclerotic heart disease (4). Angina pectoris is a common clinical manifestation of CHD caused by an insufficient coronary blood supply, resulting in myocardial ischemia. Patients typically report symptoms such as pressure, tightness, or heaviness behind the sternum, which may radiate to the arms, shoulders, or jaw (5). According to a 2014 World Health Organization (WHO) report, approximately 54 million people worldwide have been affected by angina pectoris (6). Contributing risk factors include age, smoking, obesity, and high levels of low-density lipoprotein cholesterol (LDL-C) (7).

In Western medicine, angina pectoris is commonly treated with standard regimens including β-blockers, statins, calcium channel blockers, and long-acting nitrates (5, 8). However, these treatments often cause adverse effects such as high transaminase levels, nausea, vomiting, headache, and dizziness (9, 10). In Traditional Chinese Medicine (TCM), angina pectoris is classified under the terms “chest pain” (xiong bi) and “cardialgia” (xin tong). The onset of this condition is attributed to a variety of factors, including the invasion of external pathogens, dietary irregularities, emotional disturbances, internal injuries resulting from overexertion, and age-related constitutional weaknesses. These factors are believed to disrupt the circulation of Qi and blood, potentially leading to coronary vascular obstruction. The primary syndrome patterns associated with angina pectoris include Qi deficiency with blood stasis, Qi stagnation with blood stasis, phlegm-blood stasis congealment, and a dual deficiency of Qi and Yin. The principal therapeutic approach in TCM aims to promote blood circulation, eliminate blood stasis, unblock the meridians, and alleviate pain. Recent studies have indicated that Chinese patented medicines demonstrate significant advantages in the management of angina pectoris. For the syndrome of Qi deficiency with blood stasis, commonly utilized formulations include Tongxinluo Capsules, Shexiang Tongxin Dripping Pills, and Qishen Yiqi Dripping Pills (11–13). In cases of Qi stagnation with blood stasis, Compound Danshen Dripping Pills are frequently employed (14). In the context of phlegm and blood stasis interlocking syndrome, Danlou Tablets and Guanxinsutong Capsules are frequently utilized (15, 16). For cases involving dual deficiency of Qi and Yin, Wenxin Granules have been extensively recommended (17). Consequently, the promotion of blood circulation and the removal of blood stasis have been identified as fundamental therapeutic strategies for managing angina pectoris associated with coronary heart disease. When compared to the use of conventional Western medicine alone, the integration of oral Chinese patented medicines with standard Western therapies has demonstrated a significant reduction in the frequency of angina episodes, alleviation of anginal symptoms, improvement in electrocardiogram results, regulation of blood lipid levels, and reduction in blood viscosity (11, 17, 18).

Overview of reviews is a method of systematically collecting and evaluating systematic reviews or meta-analyses of a specific field of research. This approach provides a comprehensive summary of evidence, offering higher-level evidence that integrates and evaluates existing findings to improve the reliability and applicability of evidence (19). Systematic reviews have been increasingly used in traditional Chinese medicine thanks to the advancement of evidence-based medicine (20–24). Several systematic reviews confirmed that the combined use of conventional Western medicines with Chinese patented oral medicines that promote blood circulation and remove blood stasis used as adjunctive therapies for angina pectoris is more effective and safer than the exclusive use of conventional Western medicines. However, the methodological quality of existing studies exhibits inconsistency, with substantial variation in the level of evidence. Therefore, a systematic review is necessary to re-evaluate the available findings and assess the quality of the evidence. The objectives and significance of the present study are outlined in three primary aspects: Initially, the methodological quality of systematic reviews examining the use of oral Chinese patented medicines that enhance blood circulation and alleviate blood stasis as adjunctive treatments for angina pectoris was assessed utilizing the AMSTAR-2 tool (25). This evaluation sought to identify methodological deficiencies and promote advancements in the design and implementation of future reviews, thereby contributing to the elevation of evidence-based research standards within TCM. Subsequently, the reporting quality of the included systematic reviews was evaluated in accordance with the PRISMA 2020 guidelines (26). This initiative aimed to standardize reporting practices, enhance transparency, and improve the reliability and reproducibility of systematic reviews concerning angina pectoris. Finally, a comprehensive analysis of efficacy and safety outcomes was performed, and the certainty of the evidence was appraised using the GRADE approach. This assessment aimed to elucidate the strength of evidence supporting the combined use of Chinese patented medicines and conventional Western therapies in the management of angina pectoris. This study offers significant insights for clinical practice and the formulation of treatment guidelines, while simultaneously identifying existing evidence gaps and contributing to the design of future high-quality clinical trials.

## 2 Materials and methods

### 2.1 Search strategy

A systematic search was performed using eight databases, such as CNKI, Wanfang, VIP, CBM, PubMed, the Cochrane Library, Embase, and Web of Science, to identify systematic reviews on Chinese patented oral medicines promoting blood circulation and removing blood stasis for the treatment of angina pectoris. The search covered publications from the inception of each database to July 18, 2024. The search terms were developed using a combination of MeSH terms and free-text words, with the primary terms being “coronary heart diseases”, “angina pectoris”, “Chinese medicine”, “Promoting Blood Circulation and Removing Blood Stasis”, “Huoxue Huayu”, “Chinese Patented Oral Medicines”, “systematic review”, and “meta-analysis”.

Taking PubMed as an example, the specific search formula was the following:

(“Chinese medicine”[Title/Abstract] OR “Huoxue Huayu”[Title/Abstract] OR “Promoting Blood Circulation and Removing Blood Stasis”[Title/Abstract] OR “Chinese Patented Oral Medicines”[Title/Abstract] AND [“meta-analysis”[Title] OR “systematic review”[Title]] AND [“coronary heart diseases”[Title] OR “Angina Pectoris”[Title]].

### 2.2 Inclusion and exclusion criteria

#### 2.2.1 Inclusion criteria

Study type: meta-analyses based on randomized controlled trials (RCTs).

Participants: patients diagnosed with angina pectoris, regardless of age, gender, ethnicity, or disease duration.

Interventions: the treatment group received Chinese patented oral medicine promoting blood circulation and removing blood stasis, combined with conventional Western medicine. The classification of Chinese medicines followed the standards published in the Pharmacopoeia of the People's Republic of China (2020 edition). The control group received only the conventional Western medicine treatment.

Outcomes: the main outcomes included efficacy in treating angina pectoris (A comprehensive evaluation was performed based on indicators such as angina attack frequency, duration, symptom scores, and severity.), overall efficacy (A comprehensive evaluation was performed based on indicators such as clinical efficacy, physical signs, and the overall effective rate.), electrocardiogram (ECG), frequency of angina attacks, duration of angina attacks, incidence of cardiovascular events, total cholesterol (TC), triglycerides (TG), low-density lipoprotein cholesterol (LDL-C), high-density lipoprotein cholesterol (HDL-C), high-sensitivity C-reactive protein (hs-CRP), interleukin-6 (IL-6), and dosage of nitroglycerin.

#### 2.2.2 Exclusion criteria

Study protocols or preclinical animal studies. Network meta-analyses, overview of reviews, or narrative systematic reviews. Conference abstracts, newspaper articles, or research updates. Patients with co-morbid conditions or combined diseases. Incomplete data, duplicate publication, or unavailable full texts. Interventions with more than one Chinese patent medicine.

### 2.3 Literature screening and data extraction

Two researchers independently evaluated the titles and abstracts of the collected studies to identify the eligible ones. Duplicates were removed using NoteExpress V3.9.0. software. Discrepancies during the evaluation were resolved through discussion, with the inclusion of a third researcher to resolve potential disagreements.

Data extraction was performed using a pre-designed Excel template. Two researchers independently extracted key information, including the first author, publication year, treatment and control interventions, outcomes, used databases, number of RCTs, total sample size, RCT quality assessment tools, adverse events, and study conclusions. Discrepancies in data extraction were resolved through discussion or third-party adjudication.

### 2.4 Methodological and reporting quality assessment

In this study, the methodological and reporting quality of the selected systematic reviews was assessed independently by two researchers. Discrepancies or disagreements during the evaluation were resolved through discussion until reaching a consensus. The methodological quality of the included studies was evaluated using the AMSTAR-2 tool, which consists of 16 assessment items. Among them, items 2, 4, 7, 9, 11, 13, and 15 were designated as critical items, Such as: whether a pre-study protocol was established, whether the search strategy was comprehensive, whether the exclusion criteria were provided, whether the risk of bias assessment methods were appropriate, whether the statistical analysis methods were suitable, whether the risk of bias was considered when interpreting or discussing the results, and whether the potential for publication bias and its impact on the results were assessed and discussed. The remaining items (1, 3, 5, 6, 8, 10, 12, 14, and 16) were considered non-critical (20). The evaluation result for each item was categorized as “Yes” (Y), “Partially Yes” (PY), or “No” (N), depending on whether the study met the criteria for that specific item. The overall methodological quality of each systematic review was classified as follows: high quality: at most one non-critical item not satisfied. Moderate quality: more than one non-critical item not satisfied. Low quality: one critical item not satisfied, regardless of non-critical items. Very low quality: more than one critical item not satisfied, regardless of non-critical items.

The reporting quality was assessed using the PRISMA 2020 checklist, which is composed of seven sections: title, abstract, introduction, methods, results, discussion, and other information. It encompasses a total of 27 items (42 sub-items). Item 1 evaluated the quality of the title; item 2 evaluated the quality of the abstract; items 3–4 evaluated the quality of the introduction; items 5–15 assessed the quality of the methods; items 16–22 assessed the quality of the results; item 23 evaluated the quality of the discussion; items 24–27 assessed the quality of other information. Each item was evaluated as “Yes” (Y), “Partially Yes” (PY), or “No” (N) based on whether the study met the requirements (21). This dual assessment approach ensured a comprehensive assessment of both the methodological rigor and completeness of reporting the included studies.

### 2.5 Evidence quality assessment

Two researchers independently used the GRADE pro GDT tool (https://gradepro.org/) to assess the quality of the outcomes. As regards meta-analyses based on RCTs, the initial quality of evidence was rated as “high” and downgraded based on limitations in study design, inconsistency, indirectness, imprecision, or publication bias. The final evidence quality was classified as “high”, “moderate”, “low”, or “very low”. Accuracy was ensured by assessing each outcome separately when multiple meta-analyses evaluated the same outcome.

### 2.6 Statistical analysis

A qualitative description of the basic characteristics of the included studies was provided. A descriptive analysis was performed on methodological quality, reporting quality, and evidence quality of the outcomes.

Results were visualized as follows:

(1) Bar graphs illustrating the evaluation of the methodological quality.
(2) Radar charts reporting quality analysis.
(3) Evidence plots were used to display the quality of the evidence and the effect of the treatment, incorporating the following dimensions: the *x*-axis represented the level of the evidence quality (from “high” to “very low”). The *y*-axis represented the outcome measures. The bubble size indicated the sample size, with larger bubbles corresponding to a larger study population. Bubble color indicated statistical significance, with blue representing *P* < 0.05 and orange representing *P* > 0.05.

## 3 Results

### 3.1 Literature screening results

**Figure 1:**
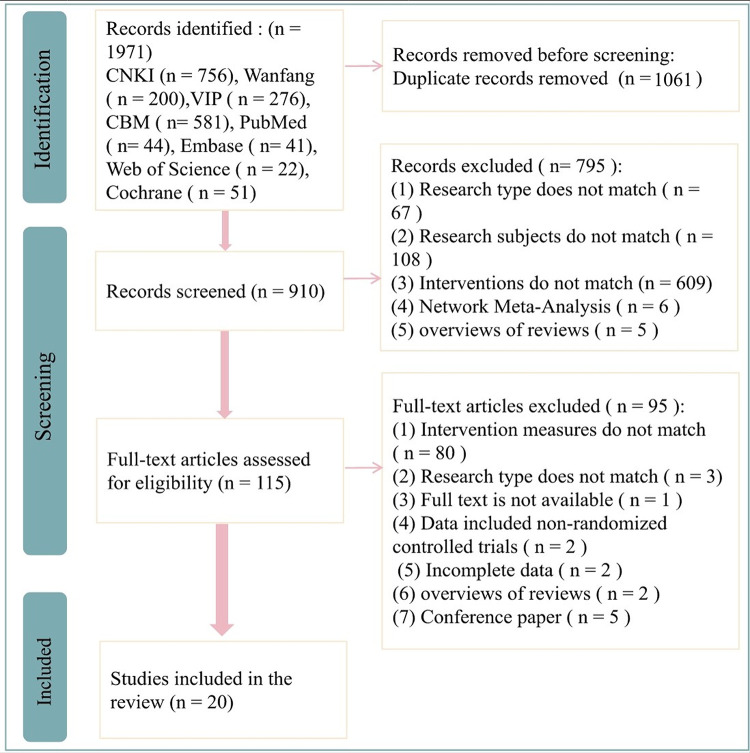
Flowchart of literature selection.

A total of 1,971 records were collected during the initial database search, but 910 unique articles remained after removing duplicates. Subsequently, 115 articles were selected for further evaluation based on a careful analysis of their titles and abstracts according to the predefined inclusion and exclusion criteria. Ninety-five articles were excluded for not meeting the inclusion criteria after a full-text examination. Finally, 20 studies (22–41) were included in this analysis. The detailed literature selection is shown in Figure 1.

### 3.2 Basic characteristics of the included studies

In this study, 20 Meta-analyses (27–46) were analyzed, which involved a total of 41,231 patients with angina pectoris. During the literature search, the most frequently used English databases were PubMed, Cochrane Library, Embase, and Medline, while the Chinese databases were CNKI, Wanfang, VIP, and CBM. In the included studies, the number of RCTs ranged from a minimum of 7 to a maximum of 59. Sample size ranged from 722 to 5,130 patients.

As regards the evaluation of RCT quality, 4 studies used the Jadad scale, 14 studies used the RoB scale, and 2 studies used both the RoB and Jadad scales. The studies involved a variety of Chinese oral medicines, including Yangxinshi Pian, Diao Xin Ke Kang Jiaonang, Xinmaitong, Tongxinluo Jiaonang, Yinxing Qon Zhi Dimian, Guanxin Danshen Dimian, Yixinshi Jiaonang, Shenshao Jiaonang, Xuezuan Xinma Ning, Mexiang Baoxin Wan, Wenxin Keli, Guanxin Ning Pian, Naoxin Tong Jiaonang, Liqi Huoxue Dimian, and Mexiang Tongxin Dimian. The outcomes assessed included the efficacy in treating angina pectoris, overall efficacy, efficacy on clinical symptom and ECG, frequency of angina attacks, duration of angina attacks, incidence of cardiovascular events, TC, TG, LDL-C, HDL-C, hs-CRP, IL-6, and dosage of nitroglycerin.

In terms of reported adverse events, 14 studies provided detailed reports. Two studies found no significant adverse reactions, while the remaining 12 reported adverse events such as digestive discomfort, dizziness, headache, and facial flushing. The basic characteristics of the included studies are listed in Table 1.

**Table 1:** Basic characteristics of the included studies.

Study	Search database	Number of included RCTs	Total sample size	Quality assessment tool	Herbal intervention	Control intervention	Outcome measures	Adverse events	Conclusion
Zhu et al. (27)	CNKI, VIP, CBM, PubMed, Cochrane Library	13	1,435	RoB	Yangxinshi Tablets combined with conventional Western medicines	Conventional Western medicine treatment: antiplatelet agents, statins, β-blockers, ACE inhibitors, or angiotensin receptor antagonists	(1), (3), (7), (8), (9), (11)	Gastric discomfort	Yangxinshi Tablets combined with conventional Western medicines were more effective
Zhou and Dai (28)	CNKI, WanFang, VIP, PubMed	13	1,024	Jadad	Yangxinshi Tablets combined with conventional Western medicines	Conventional Western medicine treatment: nitrate drugs, aspirin, statins, β-blockers	(1), (3) (11), (20)	No significant adverse reactions	Yangxinshi Tablets combined with conventional Western medicines were more effective
Zhang et al. (29)	CNKI, WanFang, VIP, CBM, Cochrane Library, Medline, EMBASE	8	722	RoB	Dioxiancao Capsules combined with conventional Western medicines	Conventional Western medicines: calcium channel blockers, nitrates	(4), (5)	Not reported	Dioxiancao Capsules combined with conventional Western medicines were more effective
Zhang et al. (30)	CNKI, WanFang, VIP, PubMed	23	2,160	Jadad; RoB	Yangxinshi Tablets combined with conventional Western medicines	Conventional Western medicines: antiplatelet agents, statins, β-blockers	(1), (2), (3), (11), (19), (20), (21)	No significant adverse reactions	Yangxinshi Tablets combined with conventional Western medicines were more effective
Yang et al. (31)	CNKI, WanFang, VIP, PubMed	8	985	RoB	Xinmaitang combined with conventional Western medicines	Conventional Western medicines	(2), (15), (22)	Not reported	Xinmaitang combined with conventional Western medicines was more effective
Wang and Kuang (32)	Cochrane Library, Cochrane Cardiovascular Group, PubMed, EMBASE, MEDLINE, WanFang, Tsinghua Tongfang Database, VIP	21	2,187	RoB	Tongxinluo Capsules combined with conventional Western medicines	Conventional Western medicines	(1), (3)	Mild epigastric pain or nausea, facial flushing	Tongxinluo Capsules combined with conventional Western medicines were more effective
Wang et al. (33)	CNKI, Wanfang, SinoMed, PubMed, Cochrane Library, EMBASE	10	1,186	RoB	Ginkgo Ketone Ester Drops combined with conventional Western medicines	Conventional Western medicines	(2), (3), (4), (5), (23)	Facial flushing, palpitations, hypotension, mild dizziness, shortness of breath, nausea, vomiting, cough	Ginkgo Ketone Ester Drops combined with conventional Western medicines were more effective
Wang et al. (34)	CNKI, VIP, SinoMed, Chinese Medicine Journal Database, WanFang	22	2,137	RoB; Jadad	Guanyinsan Capsules combined with conventional Western medicines	Conventional Western medicines	(1), (3), (4), (6), (11), (12), (13), (14), (15), (16), (17)	Rash, gastric discomfort	Guanyinsan Capsules combined with conventional Western medicines were more effective
Tang et al. (35)	CNKI, VIP, WanFang, Medline, EMBASE	11	1,827	Jadad	Yixinsu Capsules combined with conventional Western medicines	Conventional Western medicines: nitrate drugs, statins, β-blockers	(1), (3)	Mild increase in ALT	Yixinsu Capsules combined with conventional Western medicines were more effective
Sun et al. (36)	CNKI, Wanfang, VIP, SinoMed, PubMed, Cochrane Library, EMBASE	12	1,128	RoB	Shenshao Capsules combined with conventional Western medicines	Conventional Western medicines: antiplatelet drugs, β-blockers, statins, nitrate drugs	(2), (3), (4), (5)	Diarrhea, nausea, constipation, headache, facial flushing, gastric discomfort	Shenshao Capsules combined with conventional Western medicines were more effective
Ren et al. (37)	CNKI, Wanfang, VIP, PubMed, Medline	7	860	Jadad	Xueshuan Xinmaining combined with conventional Western medicines	Conventional Western medicines	(1), (3)	Dizziness, chest tightness, palpitations, itching rash, nausea, vomiting, abdominal pain, diarrhea	Xueshuan Xinmaining combined with conventional Western medicines was more effective
Liu et al. (38)	CNKI, WanFang, VIP, CBM, Cochrane Library, PubMed	37	4,104	RoB	Shexiang Baoxin Pills combined with conventional Western medicines	Conventional Western medicines: oral anticoagulants, nitrate drugs, CCBs	(2), (3), (4), (5)	Not reported	Shexiang Baoxin Pills combined with conventional Western medicines were more effective
Liu et al. (39)	CNKI, Wanfang, SinoMed, VIP, PubMed, Cochrane Library, EMBASE, Web of Science	59	4,870	RoB	Wenxin Granules combined with conventional Western medicines	Conventional Western medicines: antiplatelet drugs, RAS inhibitors, lipid-regulating drugs, β-blockers, calcium channel blockers, nitrate drugs, metabolic drugs	(3), (4), (5)	Gastrointestinal reactions, dizziness, headache, rash, facial flushing, fatigue, hypotension	Wenxin Granules combined with conventional Western medicines were more effective
Li et al. (40)	CNKI, Wanfang, SinoMed, VIP, PubMed, Cochrane Library, EMBASE	18	2,281	RoB	Guanxinning Tablets combined with conventional Western medicines	Conventional Western medicines: β-blockers, antiplatelet drugs, lipid-regulating drugs, nitrate drugs	(1), (3), (6), (11), (18), (24)	Not specifically reported	Guanxinning Tablets combined with conventional Western medicines were more effective
Li et al. (41)	CNKI, Wanfang, SinoMed, VIP, PubMed, Cochrane Library	13	1,386	Jadad	Guanxinning Tablets combined with conventional Western medicines	Conventional Western medicines	(2), (3), (7), (9), (10), (11), (18)	Not specifically reported	Guanxinning Tablets combined with conventional Western medicines were more effective
Li et al. (42)	CNKI, Wanfang, VIP, PubMed, Cochrane Library, EMBASE, Web of Science	44	5,130	RoB	Naoxintong Capsules combined with conventional Western medicines	Conventional Western medicines: anticoagulants, antihypertensive drugs, statins, nitroglycerin	(1), (2), (3)	Epigastric discomfort, gastric discomfort, dizziness, headache, facial flushing, nasal bleeding, gum bleeding	Naoxintong Capsules combined with conventional Western medicines were more effective
Han et al. (43)	CNKI, Wanfang, SinoMed, VIP, PubMed, Cochrane Library, EMBASE, Web of Science	9	982	RoB	Liqi Huoxue Drops combined with conventional Western medicines	Conventional Western medicines	(1), (2), (4), (11), (12), (13), (19)	Gastrointestinal discomfort, gastric discomfort, dizziness, headache, rash	Liqi Huoxue Drops combined with conventional Western medicines were more effective
Feng et al. (44)	CNKI, CSOD, CCD, CBM, PubMed, Cochrane Library, EMBASE	26	4,380	RoB	Naoxintong Capsules combined with conventional Western medicines	Conventional Western medicines	(1), (2), (3), (7), (8), (9), (10, (14), (15)	Gastrointestinal reactions	Naoxintong Capsules combined with conventional Western medicines were more effective
Du et al. (45)	CNKI, Wanfang, SinoMed, VIP, PubMed	11	1,075	RoB	Shexiang Tongxin Drops combined with conventional Western medicines	Conventional Western medicines: β-blockers, nitrate drugs, calcium channel blockers (CCBs)	(4), (5)	Not reported	Shexiang Tongxin Drops combined with conventional Western medicines were more effective
Cao et al. (46)	CNKI, Wanfang, SinoMed, VIP, Cochrane Library, EMBASE, MEDLINE	13	1,372	RoB	Wenxin Granules combined with conventional Western medicines	Conventional Western medicines	(2)	Nausea, vomiting, dizziness, diarrhea, abdominal distension	Wenxin Granules combined with conventional Western medicines were more effective

(1) Efficacy in treating angina pectoris; (2) overall efficacy; (3) electrocardiogram (ECG) efficacy; (4) frequency of angina attacks; (5) duration of angina attacks; (6) incidence of cardiovascular events; (7) total cholesterol (TC); (8) triglycerides (TG); (9) low-density lipoprotein (LDL); (10) high-density lipoprotein cholesterol (HDL-C); (11) high-sensitivity C-reactive protein (hs-CRP); (12) interleukin-6 (IL-6); (13) interleukin-18 (IL-18); (14) whole blood viscosity; (15) plasma viscosity (PV); (16) hematocrit; (17) fibrinogen; (18) endothelin-1 (ET-1); (19) endothelial function marker nitric oxide (NO); (20) left ventricular ejection fraction (LVEF); (21) N-terminal pro b-type natriuretic peptide (NT-proBNP); (22) myeloperoxidase (MPO); (23) dosage of nitroglycerin; (24) duration of exercise.

### 3.3 Evaluation of the methodological quality of the included studies

**Figure 2:**
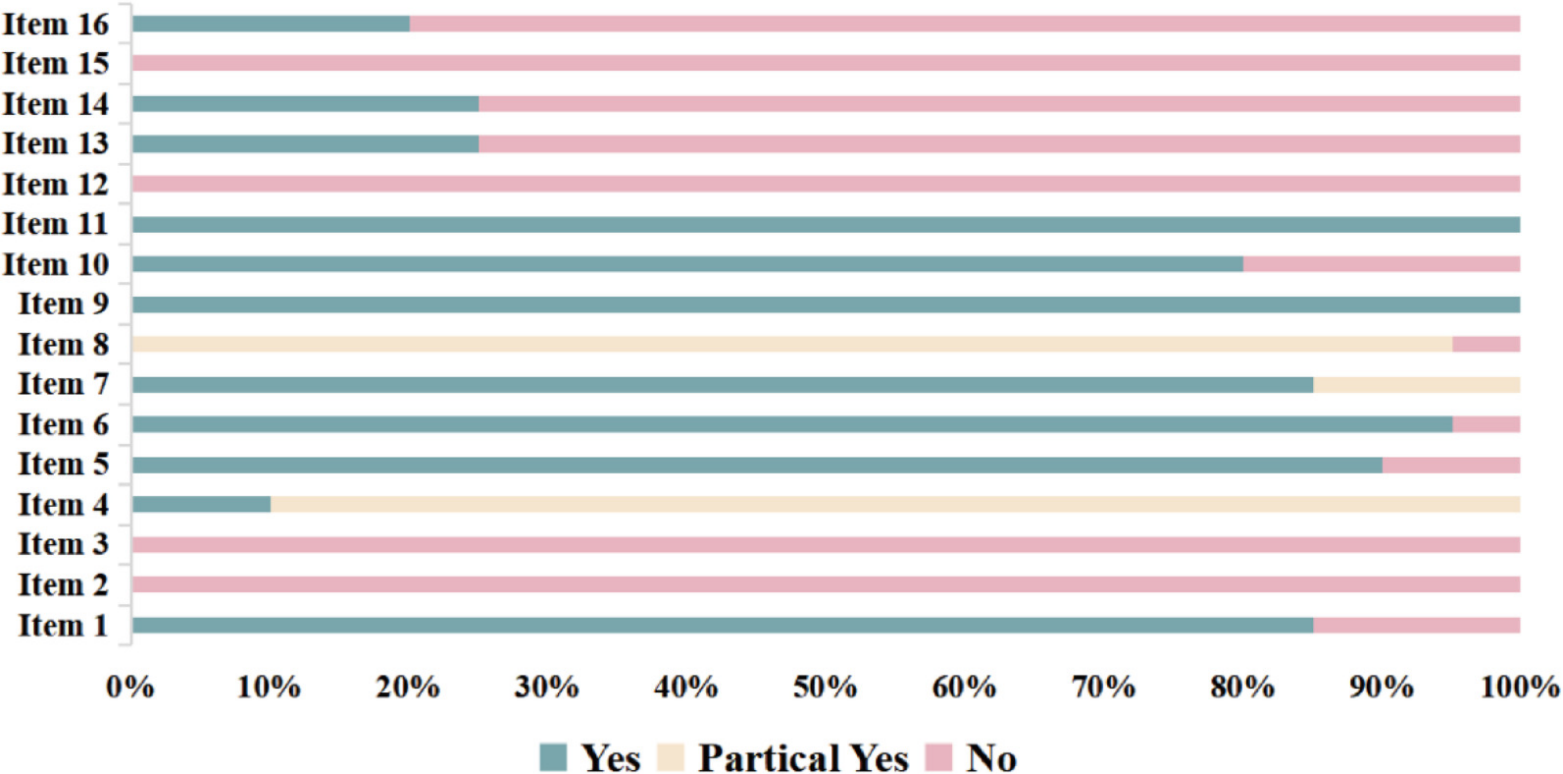
Assessment of the methodological quality.

AMSTAR-2 was used to evaluate the methodological quality of 20 studies/meta-analyses, with the results shown in Figure 2. The compliance rates for items 2, 3, and 12–16 were generally low (≤50%). No study (27–46) provided study protocols or registration details (item 2), explained the rationale for the inclusion of specific study types (item 3), or assessed the risk of bias from individual studies on the meta-analysis results (item 12). Additionally, 15 studies (27–30, 32–35, 37, 41–46) did not assess the impact of risk of bias on the results (item 13), 15 studies (28, 30, 31, 33–35, 37–45) did not adequately discuss the heterogeneity among studies (item 14), 17 studies (27, 28, 30–38, 41–46) did not sufficiently assess potential publication bias or discuss its impact, while three studies (29, 39, 41) addressed the issue but did not discuss its effects (item 15). Furthermore, 16 studies (27–29, 31–34, 37–41, 44–46) did not report potential conflicts of interest (item 16). Therefore, the methodological quality of all 20 studies (27–46) was rated as very low. A more detailed evaluation of the results is available in the Supplementary Materials.

### 3.4 Reporting quality assessment of the included studies

**Figure 3:**
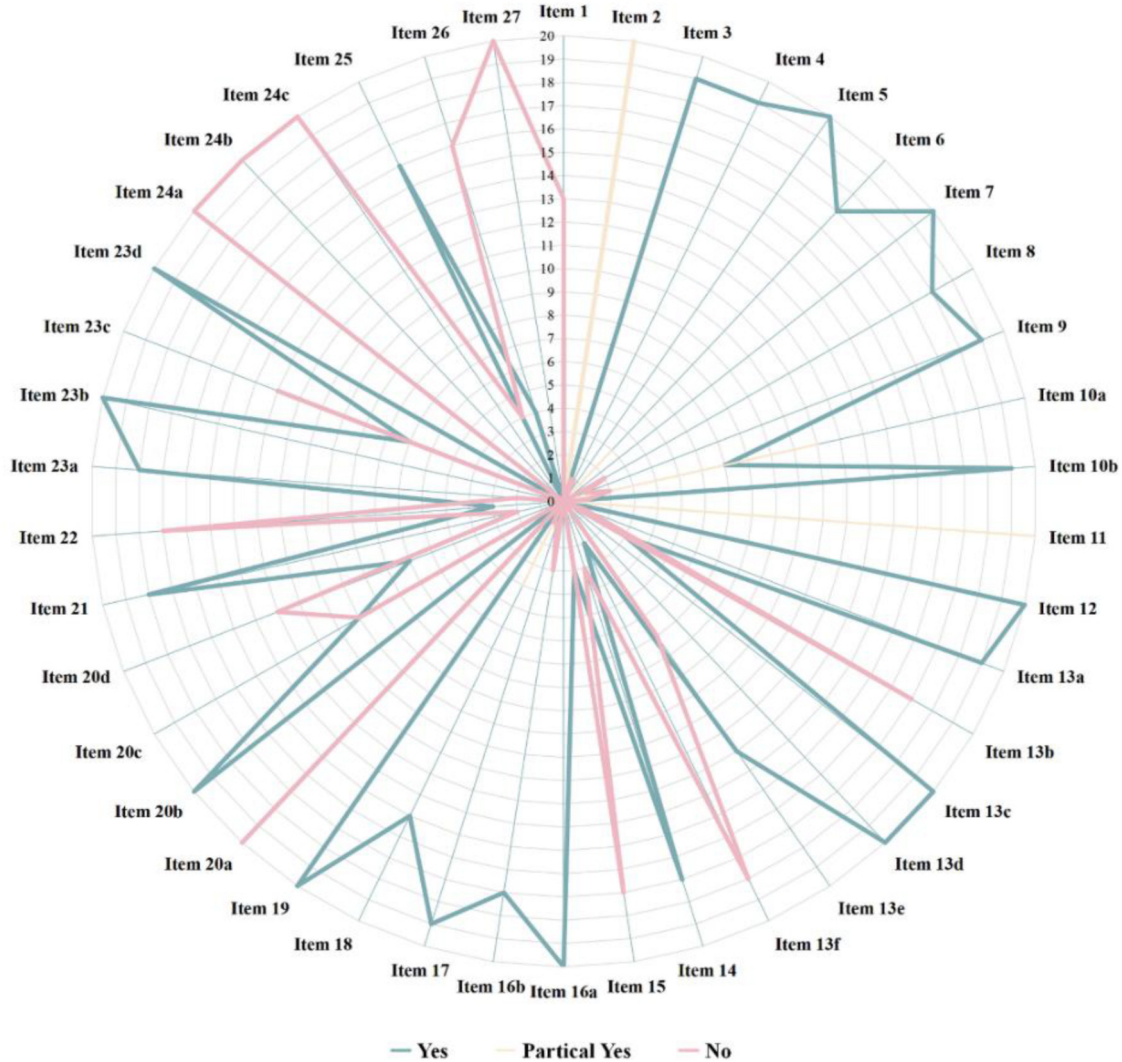
Assessment of the reporting quality.

The reporting quality of the 20 studies is shown in Figure 3. Compliance rates for items 1, 2, 10a, 11, 13b, 13f, 15, 20a, 20c, 20d, 22, 23c, 24a, 24b, 24c, 26, and 27 were generally low (≤50%). Thirteen studies (27–32, 34–36, 39–41, 43) did not clearly indicate their titles as systematic reviews (item 1). All studies (27–46) had an incomplete abstract (item 2). Two studies (41, 45) did not specify the outcome measures required for data collection, and 11 studies (27–31, 33, 34, 38, 39, 43, 45) did not clearly outline the methods used for the outcome measurement (item 10a). As regards item 11, the reporting was rated as unclear for all studies: 9 studies (27–29, 32, 36, 38, 43–45) did not mention the automated tools used for the assessment of risk of bias, three studies (31, 39, 42) did not clearly state the number of evaluators or their independence during risk assessment, and 8 studies (30, 33, 34, 35, 37, 40, 41, 46) failed to specify both the automated tools and evaluator independence. Seventeen studies (27–32, 34, 35, 38–46) did not describe whether data were pre-processed before pooling (item 13b). Eighteen studies (27–36, 38–41, 43–46) did not provide details on the assessment of sensitivity analyses to evaluate the stability of pooled results (item 13f). Seventeen studies (27–35, 37, 38, 41–46) did not describe the methods used to grade the quality of evidence (item 15). No study (27–46) explained the characteristics of each pooled result or discussed the risk of bias among them (item 20a). Ten studies (28, 31, 32, 35, 40–44, 46) did not explore potential sources of heterogeneity among them (item 20c). Thirteen studies (27–33, 35, 36, 40, 41, 44, 46) did not present the results of sensitivity analysis (item 20d). Seventeen studies (27–35, 37, 38, 41–46) did not present the results of quality grade assessment for each outcome indicator (item 22). Thirteen studies (27–29, 32–34, 36–38, 41, 44–46) did not discuss the limitations of the study process (item 23c). No study (27–46) provided the registration information (item 24a). No study (27–46) provided a pathway or statement explaining that no protocol was obtained (item 24b). No study (27–46) described or explained the modifications to registered content or protocol information (item 24c). Sixteen studies (27–29, 31–34, 37–42, 44–46) did not declare potential conflicts of interest (item 26). No study (27–46) provided information such as data extraction forms, data from the included studies, data for analysis, and data analysis code (item 27). The results of reporting quality assessment of the included studies are available in the Supplementary Materials.

### 3.5 Evidence quality assessment of the included studies

The GRADE method was used to assess the quality of evidence for each outcome indicator. The results showed that the evidence quality for 16 outcome indicators was rated as “moderate,” 29 as “low,” and 46 as “very low.” The primary reasons of this poor quality included (1) a poor methodological quality of the included RCTs in the meta-analyses, which led to a risk of bias; (2) heterogeneity with *I*^2^ > 50% and *P* < 0.01; (3) small sample sizes (<400 for continuous variables or <300 for dichotomous variables) that led to imprecision; (4) small sample size with positive results, which might indicate publication bias. The results of the detailed GRADE evidence quality assessment are available in the Supplementary Materials.

### 3.6 Efficacy assessment

**Figure 4:**
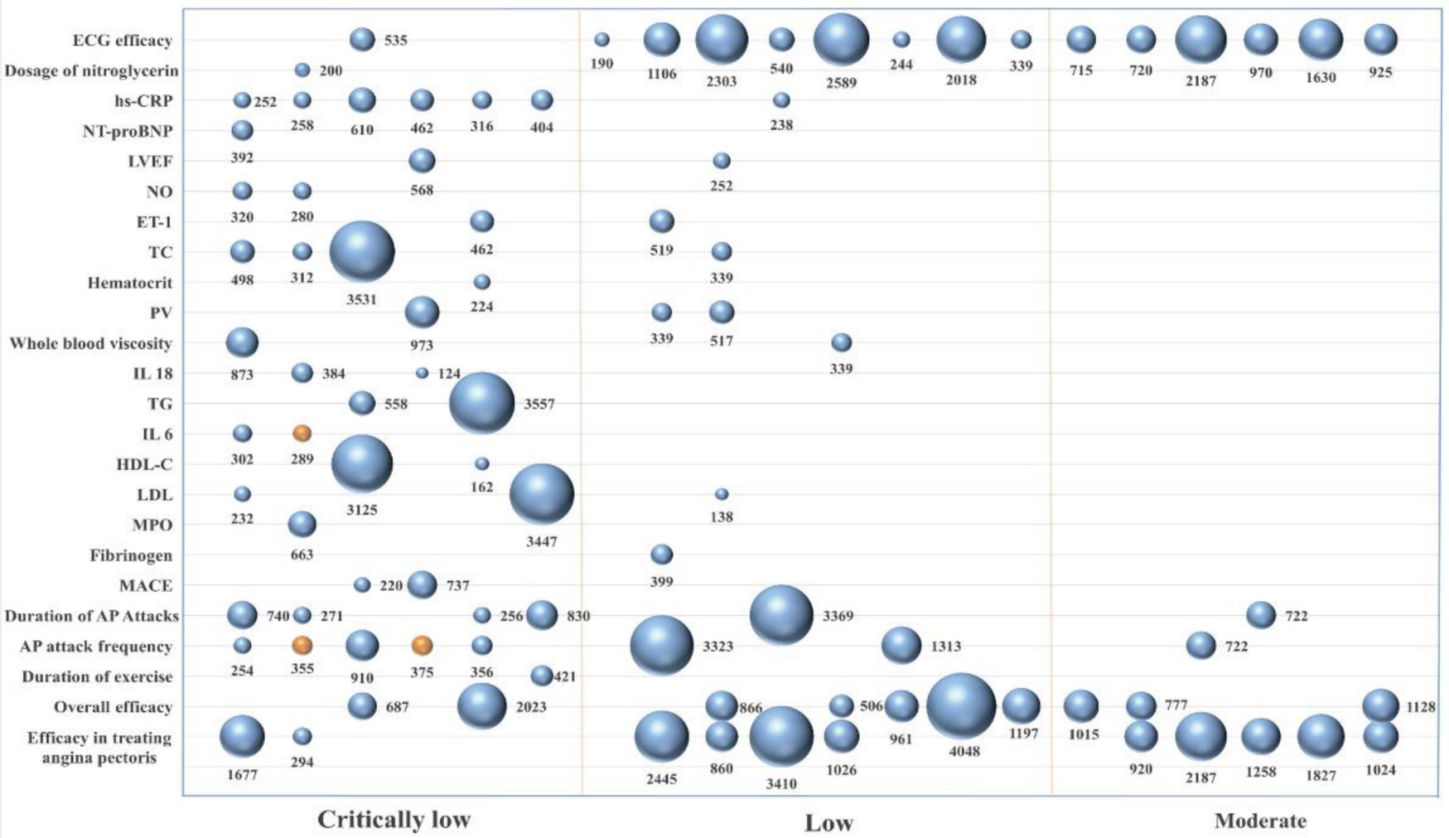
Chart of the outcome indicators.

A total of 25 outcome indicators were analyzed comparing Chinese patented oral medicines for blood-activating and stasis-dispelling combined with routine Western medicines with routine Western medicine treatment alone. The evidence quality, sample size, and *P* values for each outcome indicator are shown in Figure 4. As regards the improvement in treating angina pectoris, 11 studies (27, 28, 30, 32, 34, 35, 37, 40, 42–44) showed higher efficacy using the combination of the two treatments. As regards the improvement of the overall efficacy, 10 studies (30, 31, 33, 36, 38, 41–44, 46) indicated a higher efficacy of the combined treatment. As regards the improvement of exercise duration, 1 study (40) indicated a higher efficacy of the combined treatment. As regards the reduction of the frequency of angina attacks, 6 studies (29, 33, 36, 38, 39, 45) showed a higher efficacy of the combined treatment (*P* < 0.05), while 2 studies (34, 36) indicated no significant difference compared to the effect of routine Western medicine treatment (*P* > 0.05). As regards the reduction of the duration of angina attacks, 6 studies (29, 33, 36, 38, 39, 45) showed a higher efficacy of the combined treatment. As regards lowering MACE, 2 studies (34, 40) indicated a higher efficacy of the combined treatment. As regards reducing the fibrinogen levels, 1 study (34) showed a higher efficacy of the combined treatment. As regards lowering MPO levels, 1 study (31) indicated a higher efficacy of the combined treatment. As regards lowering LDL levels, 3 studies (27, 41, 44) demonstrated a higher efficacy of the combined treatment. As regards the regulation of HDL-C levels, 2 studies (41, 44) indicated a higher efficacy of the combined treatment. As regards the regulation of IL-6 levels, 1 study (43) showed a higher efficacy of the combined treatment (*P* < 0.05), while 1 study (34) indicated no significant difference compared to routine Western medicine treatment (*P* > 0.05). As regards lowering TG levels, 2 studies (27, 44) demonstrated a higher efficacy of the combined treatment. As regards the regulation of IL-18 levels, 2 studies (41, 44) indicated a higher efficacy of the combined treatment. As regards the reduction of the whole blood viscosity, 2 studies (34, 44) showed a higher efficacy of the combined treatment. As regards lowering plasma viscosity, 3 studies (27, 34, 44) indicated a higher efficacy of the combined treatment. As regards lowering hematocrit levels, 1 study (34) showed a higher efficacy of the combined treatment. As regards lowering TC levels, 3 studies (27, 41, 44) demonstrated a higher efficacy of the combined treatment. As regards lowering ET-1 levels, 2 studies (40, 41) showed a higher efficacy of the combined treatment. As regards the improvement of the NO levels, 2 studies (30, 43) indicated a higher efficacy of the combined treatment. As regards the improvement of left ventricular ejection fraction (LVEF), 2 studies (28, 30) showed a higher efficacy of the combined treatment. As regards lowering NT-proBNP levels, 1 study (30) demonstrated a higher efficacy of the combined treatment. As regards lowering hs-CRP levels, 7 studies (27, 28, 30, 34, 40, 41, 43) showed a higher efficacy of the combined treatment. As regards reducing nitroglycerin dosage, 1 study (33) indicated a higher efficacy of the combined treatment. As regards improving ECG efficacy, 15 studies (27, 28, 30, 32–42, 44) showed a higher efficacy of the combined treatment.

## 4 Discussion

The overview of reviews is an approach designed to systematically collect and evaluate existing systematic reviews on the treatment or diagnosis of specific diseases or health conditions. This method summarizes evidence from multiple systematic reviews to provide decision makers with aggregated and actionable insights (47). An overview was performed in this work of 20 systematic reviews on Chinese patented oral medicines categorized as “activating blood and resolving stasis” for the treatment of angina pectoris. Our results indicated that the combination of Chinese patented oral medicines of the “activating blood and resolving stasis” category with standard Western medical treatments provides significant beneficial effects, including improved angina management, ameliorated electrocardiogram, reduced frequency of angina attack, and shorter episode duration. However, the quality of the evidence was generally low, highlighting the need to enhance both the methodological rigor and reporting quality of relevant systematic reviews.

### 4.1 Methodological quality of the included studies

The methodological quality of the 20 included meta-analyses in this study was rated as “critically low.” Low compliance rates were observed for items 2, 3, 12, 13, 14, 15, and 16. Specifically, no study met the items 2, 3, 12, and 15 (27–46), reflecting the lack of prospective registration and rationale for selecting study types, as well as omission of the impact of bias in individual studies on meta-analysis results, and failure to investigate or discuss the potential influence of publication bias.

Prospective registration of systematic reviews is essential for the improvement of research transparency, reduction of selective reporting, and prevention of unintentional duplication (48). Registration requires researchers to reveal detailed protocols, including study design, inclusion and exclusion criteria, search strategies, and methods for data analysis. This process ensures that research plans are clear before their start, reducing the likelihood of selective reporting or *post hoc* design adjustments. Additionally, registration minimizes duplication by allowing other researchers to identify existing reviews and avoid repetitions. Evidence suggests that registered systematic reviews have higher methodological quality than those unregistered (49). Future reviews should therefore prioritize prospective registration to align with best practices.

Although all the 20 included studies (27–46) were RCTs, the rationale used in the selection of this study type was not explained. Although RCTs are considered the gold standard in the evaluation of clinical interventions, they are not without limitations. For example, randomization does not ensure uniform distribution of variables across groups, and RCT results are often limited to the study sample, consequently limiting their generalizability. Small-sample RCTs are particularly lacking precision, and their outcomes may be less robust despite randomization (50). Systematic reviews should consider these aspects by explaining the inclusion of RCTs and discussing why non-RCTs were excluded. Furthermore, none of the included studies (27–46) evaluated the risk of bias in individual studies, such as how the selection or measurement bias influenced the meta-analysis results. Such omission may compromise the robustness of the conclusions. Researchers are advised to systematically assess and report the impact of bias on the results of their future reviews. Finally, publication bias was not addressed in any of the included studies (27–46). Publication bias distorts the results of the meta-analysis by overestimating the efficacy of an intervention, underreporting risks, or omitting critical data (51). The detection of publication bias and the use of sensitivity analysis to evaluate the effects of missing studies are essential steps for ensuring the reliability of the conclusions of the meta-analysis.

### 4.2 Reporting quality of the included studies

The reporting quality of the 20 included studies showed low compliance for items 1, 2, 10a, 11, 13b, 13f, 15, 20a, 20c, 20d, 22, 23c, 24a, 24b, 24c, 26, and 27. Among them, items 2, 20a, 24a, 24b, 24c, and 27 were uniformly rated as “N” across all studies (27–46). Major deficiencies included incomplete abstracts, omission of descriptions for characteristics and risk of bias in combined outcomes, missing registration details. Either the protocol access method was not provided, or the existence of a protocol was not specified. Failure to explain protocol modifications, and lack of disclosure regarding the accessibility of systematic review data.

Despite the reporting guidelines such as PRISMA 2020, incomplete reporting remains common in systematic reviews (52). The abstract is essential in summarizing the study and directly impacts its dissemination and influence (53). It is the first point of contact for editors, reviewers, and readers, thus providing an initial impression of the content of the article and determining its reception and readership (54). Therefore, researchers should rigorously follow PRISMA 2020 rules when drafting the abstract.

Systematic review registration improves transparency, minimizes selective reporting, and prevents redundant reviews (48). Indeed, registered reviews generally have higher methodological quality than those without registration (49). Therefore, researchers should adhere to registration protocols to increase the quality of the reviews.

Risk of bias is another critical factor potentially leading to incorrect estimations of intervention effects and increased heterogeneity among studies (55). None of the meta-analyses described the risk of bias among studies for combined outcomes, potentially compromising the reliability of the results.

The development of a protocol for a systematic review is essential for ensuring methodological rigor and reproducibility. Protocols facilitate comprehensive literature searches and the systematic integration of the results (56). Researchers should explicitly state in their reports if a protocol is unavailable. Moreover, data sharing policies encourage transparency and allow the reuse of systematic review data. Sharing such data enables the identification of errors, promotes learning of analytical methods, reduces redundant efforts, and improves the quality and efficiency of future reviews (57).

### 4.3 Efficacy and safety of Chinese patented oral medicines that activate blood circulation in treating angina pectoris

Angina pectoris is a common clinical manifestation of CHD, characterized by a pressing or heavy sensation behind the sternum, often accompanied by chest pain (58). ECG abnormalities, such as ST-segment depression or T-wave inversion, are frequently present in patients with unstable angina, providing essential diagnostic and prognostic information during episodes of this disease (59). Individuals with a history of angina pectoris are at higher risk of cardiovascular events compared to those without (60). Inflammation and lipid metabolism are the main causes of CHD pathogenesis. Inflammatory markers such as hs-CRP and IL-6 are strongly linked to CHD risk (61), while lipid abnormality, in particular the high levels of TG, TC, and LDL-C, are the cause of disease progression (62, 63). Episodes of angina pectoris impair myocardial function, as evidenced by the reduction in LVEF during ischemia or physical exertion (64). Patients with severe angina pectoris often require higher drug doses or alternative formulations of nitroglycerin to manage symptoms, although increased dosages may lead to side effects, including headaches and development of drug resistance (65).

This study indicates that the combined use of Chinese patented oral medicines for promoting blood circulation and removing blood stasis in the treatment of angina pectoris offers more significant clinical advantages compared with conventional Western medicine treatment alone. First, this combined therapy reduces the frequency of angina pectoris attacks and shortens the duration of these attacks, thereby alleviating the symptoms in patients and improving their quality of life. Second, combined therapy improves the ECG in angina pectoris patients, particularly showing significant ameliorations in abnormalities such as ST-segment depression and T-wave inversion. The results also suggest that the combined therapy reduces the incidence of cardiovascular events, preventing disease progression and worsening. Additionally, the combination of Chinese patented oral medicines and conventional Western medicine improves inflammatory conditions, such as lowering hs-CRP levels and regulating IL-6 and IL-8 levels. In terms of lipid regulation, the study shows that this combined therapy lowers blood lipid levels, including TG, TC, and LDL-C levels. Moreover, the combined therapy enhances left ventricular ejection fraction, thus improving cardiac pumping function and overall blood perfusion. It also inhibits platelet aggregation and reduces the use of nitroglycerin.

Beyond Chinese patented medicines, a variety of natural products have shown promising cardioprotective potential. Their mechanisms of action encompass a broad spectrum of biological processes, including antioxidant, anti-inflammatory, metabolic regulatory, and anti-fibrotic effects. These natural agents are typically characterized by their multi-target and multi-pathway activities. For example, Dioscorea, Salvia miltiorrhiza, Astragalus membranaceus, Rheum palmatum, Codonopsis pilosula, Panax notoginseng, Scutellaria baicalensis, and Bupleurum chinense have been investigated for their cardiovascular benefits (66–73). Owing to their complex chemical compositions and diverse pharmacological actions, these natural products present extensive prospects for research and clinical application in the prevention and treatment of cardiovascular diseases.

As regards safety, 12 studies reported adverse reactions such as gastrointestinal discomfort, dizziness, headaches, and facial flushing. However, since the interventions were a combination of Chinese patented medicines with conventional Western therapies (e.g., statins and calcium channel blockers), it is unclear whether these reactions were caused by the Chinese patented medicines, the Western medicines, or their interaction (9) (10).

Although the combination therapy appeared effective, the GRADE assessment indicated that most supporting evidence was of low or very low quality. Two primary factors contributed to this limitation:

(1) Low methodological quality in the included RCTs, with problems of inadequate randomization, poor allocation concealment, and lack of blinding.
(2) Lack of standardized methods and criteria of the measurement of the outcome indicators, such as efficacy in treating angina pectoris, overall efficacy, and efficacy on treating clinical symptoms, resulting in inconsistent definitions and standards of evaluation.

Therefore, future RCTs should adopt rigorous randomization procedures and standardized methods for measuring and evaluating outcome indicators to improve the quality and credibility of the evidence.

### 4.4 Strengths and limitations

This study possesses the following three strengths:

(1) A comprehensive search strategy was used, covering eight databases to obtain systematic reviews on the combination therapy of Chinese patented oral medicines for activating blood circulation with conventional Western medicine for treating angina pectoris, summarizing their efficacy and safety in its management.
(2) Methodological and reporting quality of the included systematic reviews were rigorously assessed using AMSTAR-2 and PRISMA 2020 standards, respectively.
(3) Evidence quality was systematically evaluated using GRADE.
(4) Evidence quality and therapeutic effects evaluated by various outcome indicators were visually represented using bubble plots to improve the clarity and interpretability.

Despite these strength points, the study has also some limitations:

① Limitations of the literature sources: Only systematic reviews and meta-analyses from Chinese databases were included, while grey and non-Chinese literature was excluded. This restriction might lead to selection bias in the literature, limit the robustness and generalizability of the results, reducing their relevance for an international audience. This makes it difficult to comprehensively reflect the progress of international research and consensus in this field. ② The factors of Traditional Chinese Medicine syndrome differentiation were not sufficiently considered: The absence of differentiation of angina pectoris syndromes in the included studies, a fundamental principle of Traditional Chinese Medicine, that is, the development of an individualized treatment plan based on the specific condition of each patient, prevented the understanding of the difference in the effects of the treatments, This limits the assessment of the precise therapeutic effects of such Chinese patent medicines and is not conducive to guiding syndrome-based treatment in the clinical practice of Traditional Chinese Medicine for the treatment of angina pectoris. ③ The methodological quality is generally low: according to the AMSTAR-2 evaluation results, the included studies have significant deficiencies in key areas such as study protocol pre-registration and risk of bias assessment. The overall low methodological quality of the included studies, including insufficient randomization and lack of blinding, raises concerns about the reliability and validity of the findings.

## 5 Conclusion

The combination of Chinese patented oral medicines for the activation of blood circulation with conventional Western medicine shows significant advantages over the use of Western medicine alone in managing angina pectoris. The main benefits include reduced attack frequency and duration, improved electrocardiogram, decreased incidence of cardiovascular events, reduced inflammation and lipid levels, improved LVEF, inhibition of platelet aggregation, and lowered nitroglycerin usage. The treatment also showed a favorable safety profile. Overall, the results of this study provide strong evidence for clinical practice and guideline development, contributing to the promotion of the integrated application of combined Traditional Chinese and Western medicine interventions in the treatment of angina pectoris.

Despite these promising results, systematic reviews on this combination therapy require improvements in methodological rigor and reporting transparency. The adhesion to AMSTAR-2 (20) and PRISMA 2020 (21) standards is essential for future evaluations. The amelioration of the design, performance, and reporting quality of primary RCTs is also essential. Future research should prioritize rigorous methodologies and standardized practices to improve reliability and clinical applicability.

## Data Availability

The raw data supporting the conclusions of this article will be made available by the authors, without undue reservation.
