# Supplementary material for: Overview of systematic reviews on Chinese patented oral medicines for promoting blood circulation and removing blood stasis combined with western medicine in the treatment of coronary heart disease angina pectoris
Source: Front Cardiovasc Med. 2025 Jun 20;12:1553735. doi: 10.3389/fcvm.2025.1553735 (PMC12226556; doi:10.3389/fcvm.2025.1553735)
Supplement: Supplementary file 1 [file Datasheet1.pdf]

MogoEdit

# CERTIFICATE OF ENGLISH EDITING

This is to certify that the manuscript entitled

***Overview of Systematic Reviews of Chinese Patented Oral Medicines for Promoting Blood Circulation and Removing Blood Stasis in the Adjuvant Treatment of Coronary Heart Disease Angina Pectoris***

commissioned to us has been carefully edited by a native English-speaking editor of MogoEdit, and the grammar, spelling, and punctuation have been verified and corrected where needed. Based on this review, we believe that the language in this paper meets academic journal requirements. Please contact us with any questions.

MOGOEDIT
MOGO INTERNET TECHNOLOGY CO., LTD.

Gang Zhang

Dr. Gang Zhang
Founder & CEO of MogoEdit

Date of Issue
December 24, 2024

**Disclaimer:** The changes in the document may be accepted or rejected by the authors in their sole discretion after our editing. However, MogoEdit is not responsible for revisions made to the document after our edit on **December 24, 2024.**

MogoEdit is a professional English editing company who provides English language editing, translation, and publication support services to individuals and corporate customers worldwide. As a company invested by the affiliate fund of Chinese Academy of Science, MogoEdit is one of the leading language editing service providers in China, whose clients come from more than 1000 universities and research institutes.
MogoEdit Website: http://www.mogoedit.com/
500+ native English editors: http://www.mogoedit.com/editors

Mogo make the world flatter

Mogo Internet Technology Co., LTD.
No. 57, 3rd Keji Road, Xi'an 710075, PR China +86 02988317483 support@mogoedit.com
