# Supplementary material for: Overview of systematic reviews on Chinese patented oral medicines for promoting blood circulation and removing blood stasis combined with western medicine in the treatment of coronary heart disease angina pectoris
Source: Front Cardiovasc Med. 2025 Jun 20;12:1553735. doi: 10.3389/fcvm.2025.1553735 (PMC12226556; doi:10.3389/fcvm.2025.1553735)
Supplement: Supplementary file 2 [file Datasheet2.pdf]

November 20, 2024 at 10:36

We now need to discuss: According to what criteria are included in the blood-activating and blood-stasis drugs?

Jiang Junjie

Proprietary Chinese medicine with the effect of promoting blood circulation and removing blood stasis, treating diseases including coronary heart disease and angina pectoris, will do

Jiang Junjie

This is the main inclusion criterion

Well. 🌹

November 20, 2024 at 10:47 PM

Xie Yanming, China Academy of Chinese Medical Sciences

Not only to evaluate the quality of the results, but also to point out how to improve

Xie Yanming, China Academy of Chinese Medical Sciences

Suggestions on improving quality

Jiang Junjie

Uh-huh, agreed 🌹 That's the point of the discussion 🫴 🫴🫴

Ok, thank you. Next, revise the manuscript according to the teacher's suggestions.

Xie Yanming, China Academy of Chinese Medical Sciences: Not only should we evaluate the quality of the results, but also point out how to improve them

Wu Hongli

The treatment of angina pectoris is not the Chinese patent medicine have the effect of activating blood stasis so what is the exclusion criteria

11:22, November 20, 2024

Initial consideration is to refer to the Pharmacopoeia of the People's Republic of China to include in the pharmacopoeia proprietary Chinese medicines with the effect of promoting blood circulation and removing blood stasis

Wu Hongli: Do all the proprietary Chinese medicines for the treatment of angina pectoris have the effect of promoting blood circulation and removing blood stasis

November 20, 2024 at 10:36

We now need to discuss: According to what criteria are included in the blood-activating and blood-stasis drugs?

Jiang Junjie

Proprietary Chinese medicine with the effect of promoting blood circulation and removing blood stasis, treating diseases including coronary heart disease and angina pectoris, will do

Jiang Junjie

This is the main inclusion criterion

Well. 🌹

November 20, 2024 at 10:47 PM

Xie Yanming, China Academy of Chinese Medical Sciences

Not only to evaluate the quality of the results, but also to point out how to improve

Xie Yanming, China Academy of Chinese Medical Sciences

Suggestions on improving quality

Jiang Junjie

Uh-huh, agreed 🌹 That's the point of the discussion

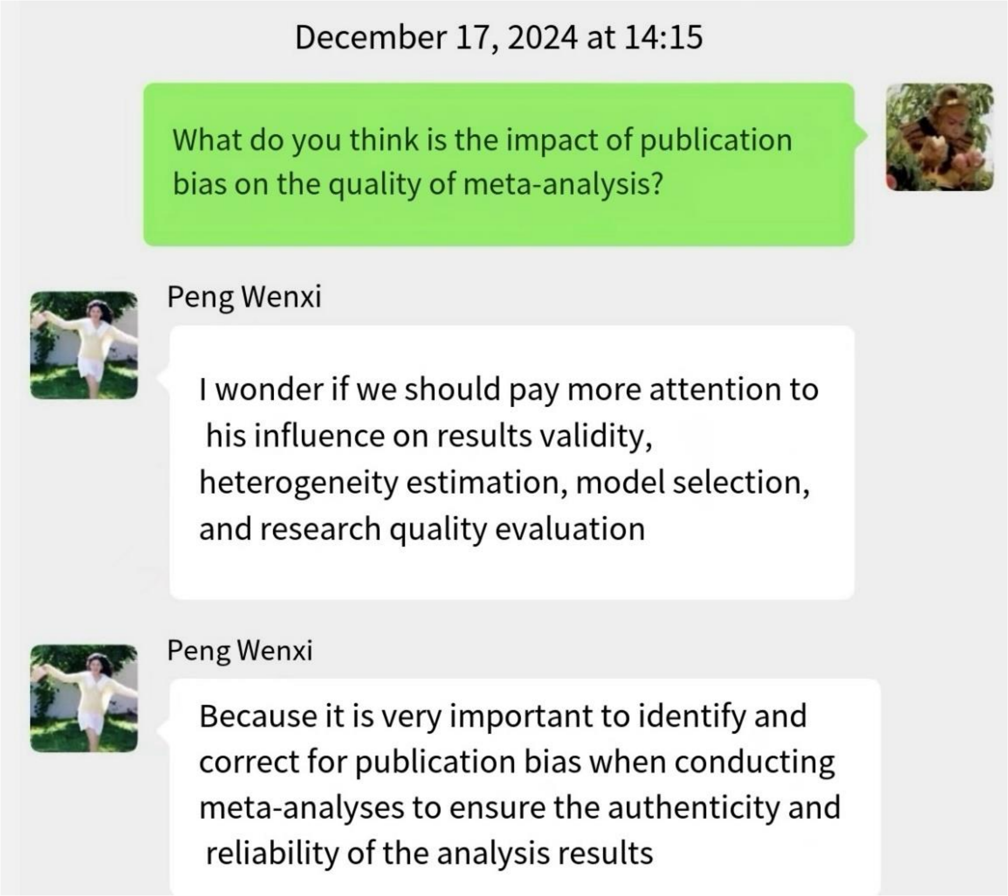
December 17, 2024 at 14:15
What do you think is the impact of publication bias on the quality of meta-analysis?
Peng Wenxi
I wonder if we should pay more attention to his influence on results validity, heterogeneity estimation, model selection, and research quality evaluation
Peng Wenxi
Because it is very important to identify and correct for publication bias when conducting meta-analyses to ensure the authenticity and reliability of the analysis results

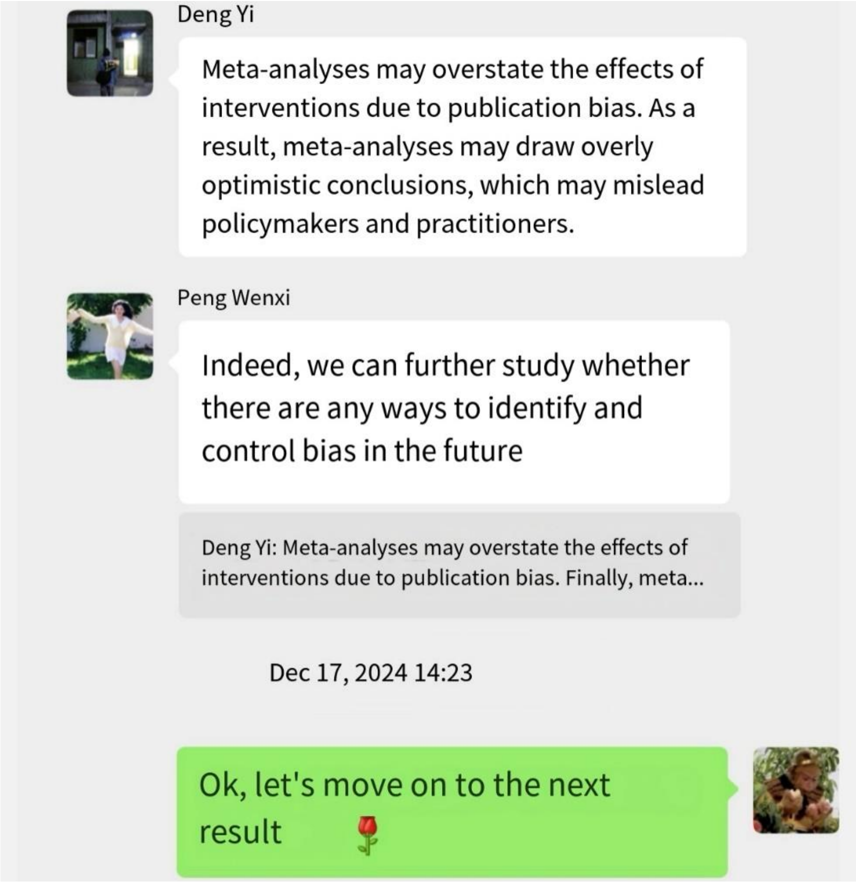
Deng Yi
Meta-analyses may overstate the effects of interventions due to publication bias. As a result, meta-analyses may draw overly optimistic conclusions, which may mislead policymakers and practitioners.
Peng Wenxi
Indeed, we can further study whether there are any ways to identify and control bias in the future
Deng Yi: Meta-analyses may overstate the effects of interventions due to publication bias. Finally, meta...
Dec 17, 2024 14:23
Ok, let's move on to the next result 🌹
