# Supplementary figures and images for: Overview of systematic reviews on Chinese patented oral medicines for promoting blood circulation and removing blood stasis combined with western medicine in the treatment of coronary heart disease angina pectoris
Source: Front Cardiovasc Med. 2025 Jun 20;12:1553735. doi: 10.3389/fcvm.2025.1553735 (PMC12226556; doi:10.3389/fcvm.2025.1553735)

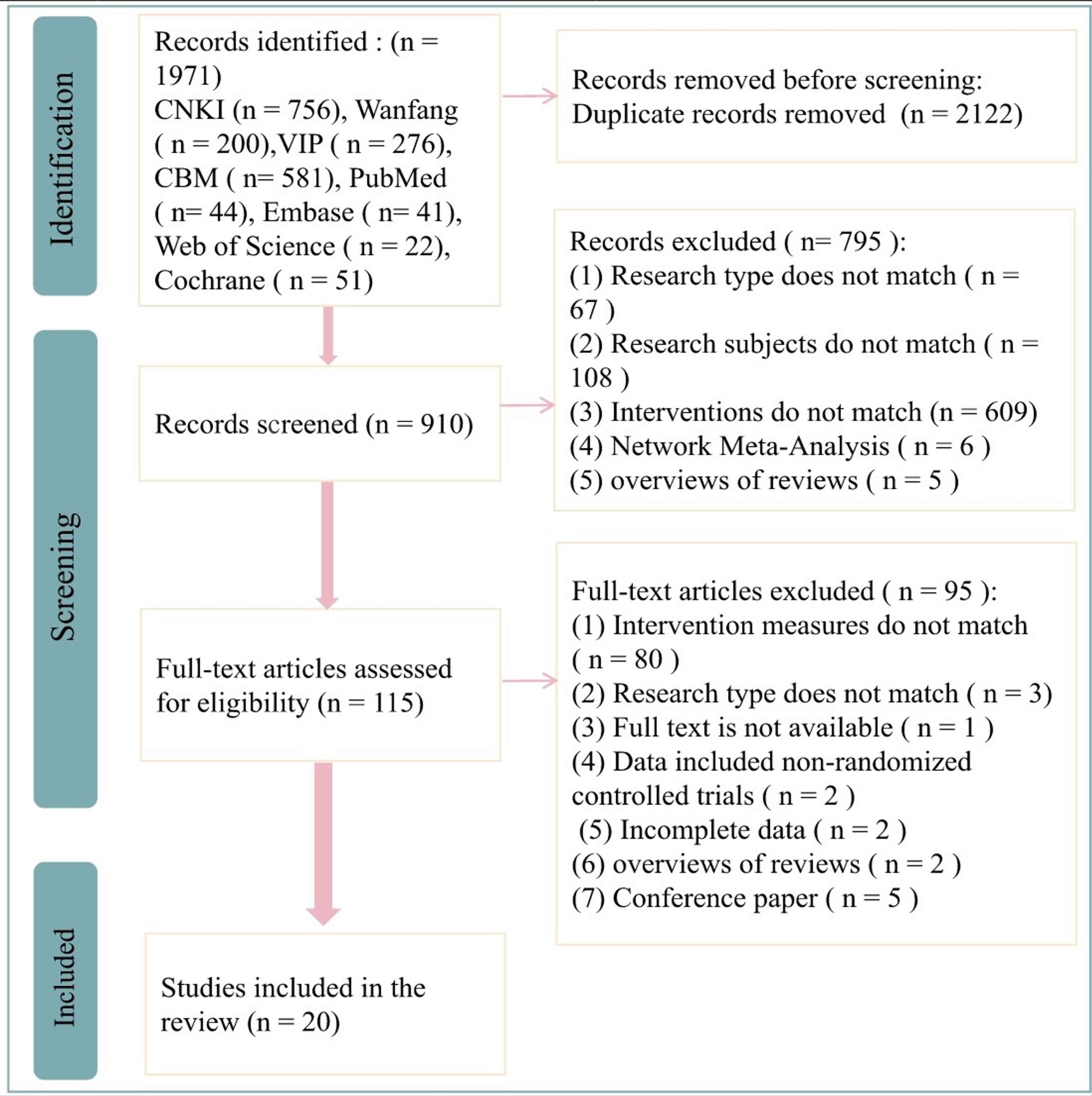

Supplement: Supplementary file 3 [file Image1.png]

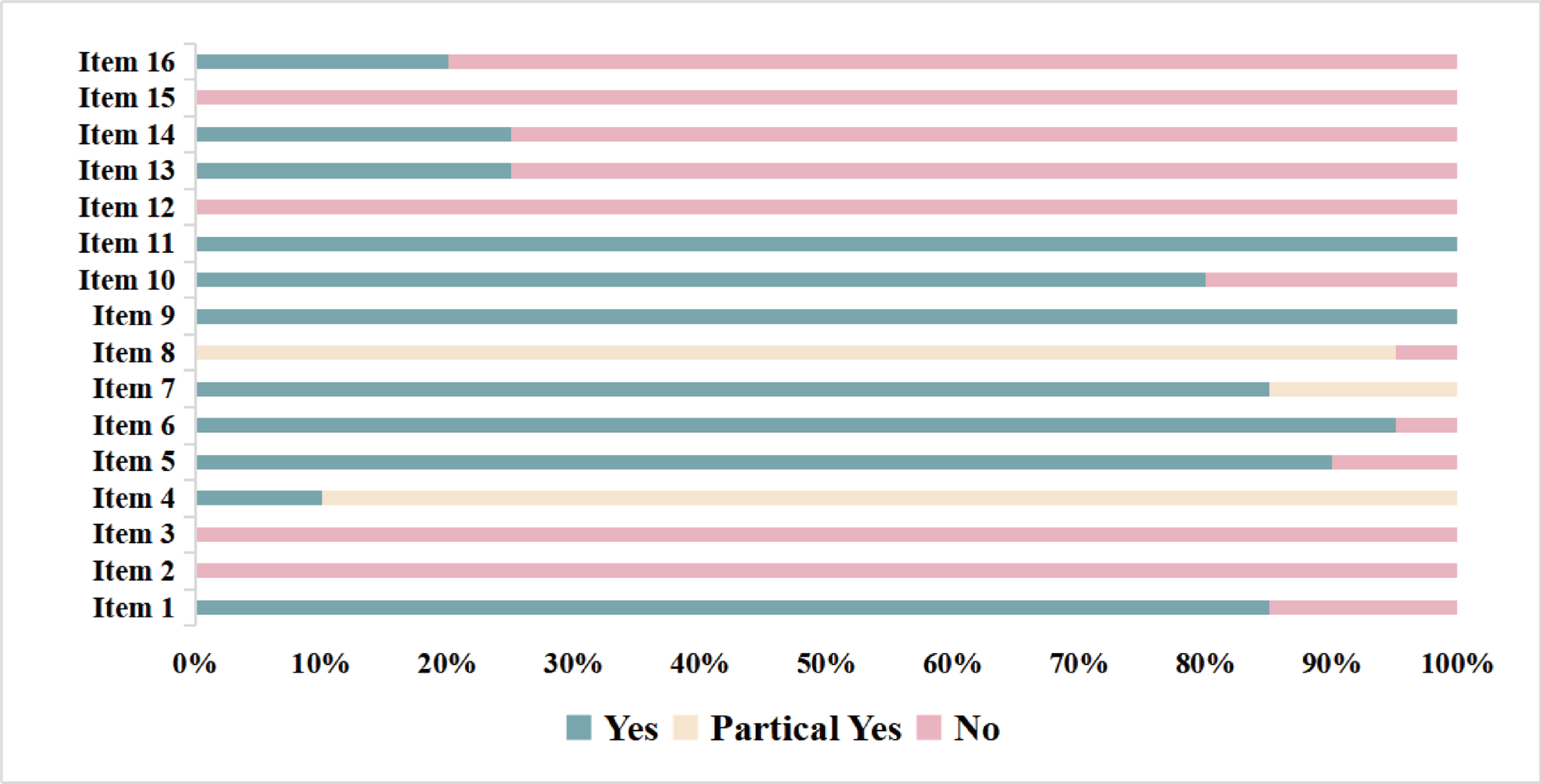

Supplement: Supplementary file 4 [file Image2.png]

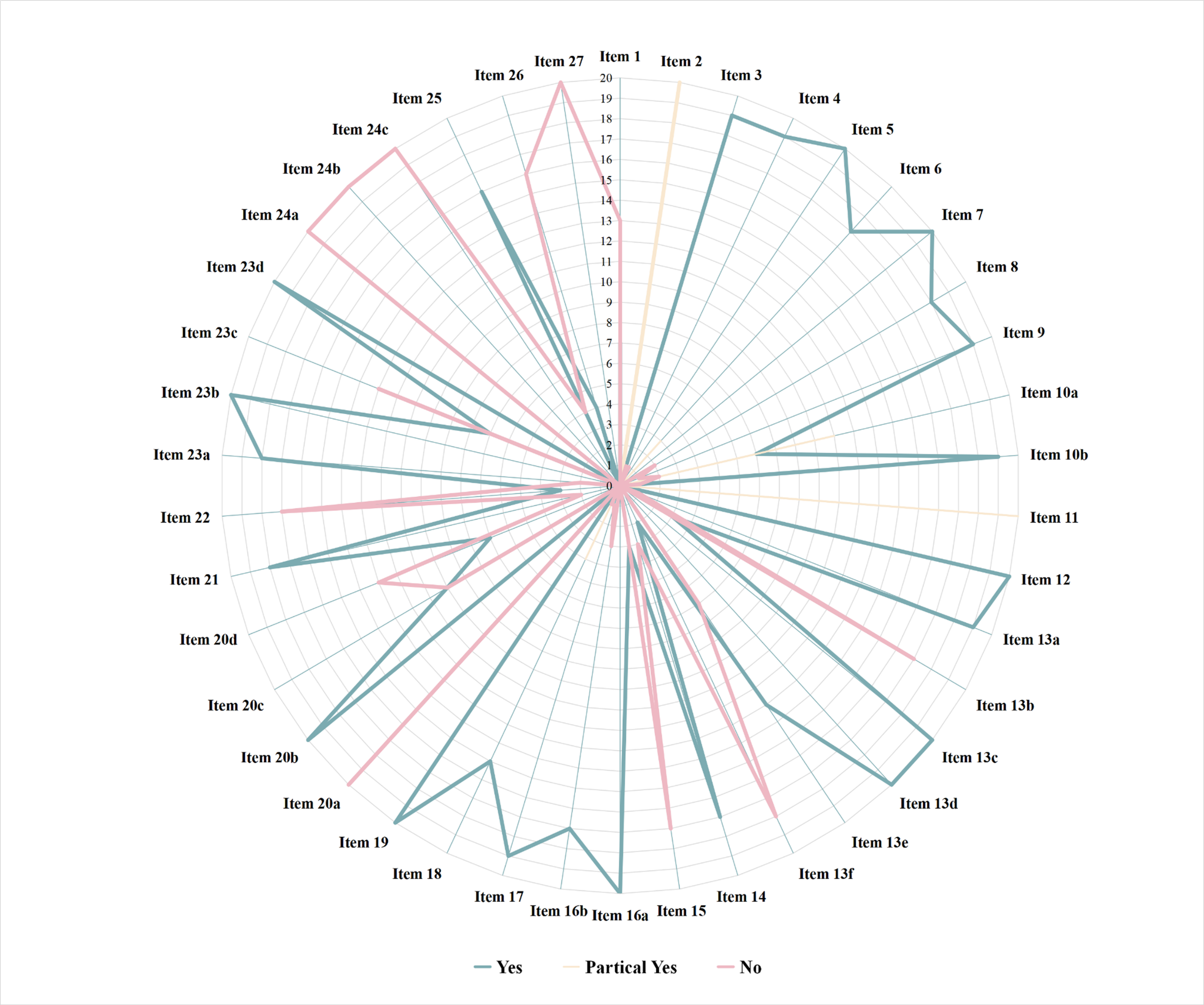

Supplement: Supplementary file 5 [file Image3.png]

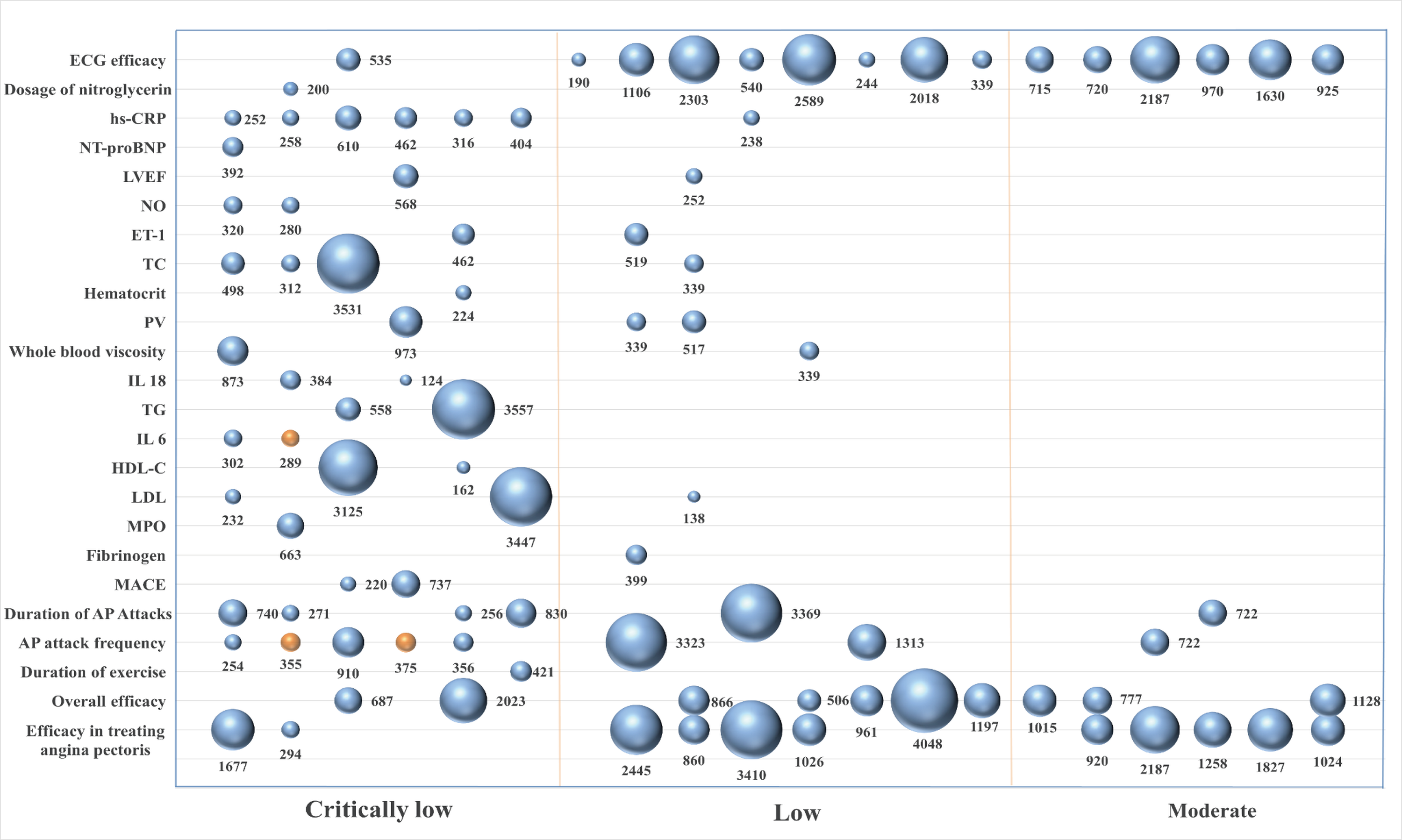

Supplement: Supplementary file 6 [file Image4.png]
