# Supplementary material for: Overview of systematic reviews on Chinese patented oral medicines for promoting blood circulation and removing blood stasis combined with western medicine in the treatment of coronary heart disease angina pectoris
Source: Front Cardiovasc Med. 2025 Jun 20;12:1553735. doi: 10.3389/fcvm.2025.1553735 (PMC12226556; doi:10.3389/fcvm.2025.1553735)
Supplement: Supplementary file 9 [file Table3.docx]

**The results of evidence quality evaluation by GRADE**

（Chinese patent medicines for promoting blood circulation and removing blood stasis vs.conventional Western medicine treatment）

| Outcomes | Study | Effect Size (95% CI) | *P* | I^2^(%) | Risk of bias | Inconsistency | Indirectness | Imprecision | Publication bias | GRADE quality |
| --- | --- | --- | --- | --- | --- | --- | --- | --- | --- | --- |
| Efficacy in treating angina pectoris | Zhu XL 2018(21) | OR:3.93(2.51,6.16) | <0.00001 | 0 | serious^a^ | not serious | not serious | not serious | none | Moderate |
|  | Zhou J 2018(22) | OR:4.12(2.89, 5.88) | <0.00001 | 0 | serious^a^ | not serious | not serious | not serious | none | Moderate |
|  | Zhang HY 2022(24) | OR:4.87(3.55, 7.08) | <0.001 | 0 | serious^a^ | not serious | not serious | not serious | publication bias strongly suspected^f^ | Low |
|  | Wang Y 2012(26) | OR:2.83(2.25, 3.55) | <0.00001 | 5 | serious^a^ | not serious | not serious | not serious | none | Moderate |
|  | Wang HJ 2020(28) | OR:3.87(2.68, 5.60) | <0.00001 | 0 | serious^a^ | not serious | not serious | not serious | none | Moderate |
|  | Tang W 2016(29) | OR:0.3(0.23, 0.40) | <0.001 | 43 | serious^a^ | not serious | not serious | not serious | none | Moderate |
|  | Ren ZX 2018(31) | OR:2.60(1.82, 3.73) | <0.00001 | 28 | serious^a^ | not serious | not serious | not serious | publication bias strongly suspected^f^ | Low |
|  | Li XY 2023(34) | RR:1.33(1.13, 1.57) | 0.0008 | 93 | serious^a^ | serious^b^ | not serious | not serious | publication bias strongly suspected^f^ | Very low |
|  | Li CX 2019(36) | RR:1.24(1.19, 1.29) | <0.00001 | 0 | serious^a^ | not serious | not serious | not serious | publication bias strongly suspected^f^ | Low |
|  | Han Y 2022(37) | RR:1.17(1.07, 1.29) | 0.001 | 0 | serious^a^ | not serious | not serious | serious^d^ | publication bias strongly suspected^e^ | Very low |
|  | Feng J 2021(38) | RR:0.43(0.36, 0.52) | <0.00001 | 0 | serious^a^ | not serious | not serious | not serious | publication bias strongly suspected^f^ | Low |
| Overall efficacy | Zhang HY 2022(24) | OR:3.58(2.53,5.06) | <0.001 | 0 | serious^a^ | not serious | not serious | not serious | publication bias strongly suspected^f^ | Low |
|  | Wang KY 2021(27) | RR:1.21(1.10, 1.33) | 0.0001 | 0 | serious^a^ | not serious | not serious | not serious | publication bias strongly suspected^f^ | Low |
|  | Liu ZC 2020(32) | RR:1.20(1.17, 1.23) | <0.00001 | 28 | serious^a^ | not serious | not serious | not serious | publication bias strongly suspected^f^ | Low |
|  | Cao WZ 2015(40) | OR:4.57(3.33, 6,28) | <0.00001 | 0 | serious^a^ | not serious | not serious | not serious | none | Moderate |
|  | Yang T 2022(25) | OR:4.98(3.02, 8.21) | <0.00001 | 0 | serious^a^ | not serious | not serious | not serious | none | Moderate |
|  | Sun H 2024(30) | RR:41.20(1.15, 1.26) | <0.00001 | 13 | serious^a^ | not serious | not serious | not serious | none | Moderate |
|  | Li L 2022(35) | OR:4.11(3.02, 5.60) | <0.00001 | 0 | serious^a^ | not serious | not serious | not serious | publication bias strongly suspected^f^ | Low |
|  | Li CX 2019(36) | RR:1.20(1.15, 1.25) | <0.00001 | 52 | serious^a^ | serious^b^ | not serious | not serious | publication bias strongly suspected^f^ | Very low |
|  | Han Y 2022(37) | RR:1.20(1.12, 1.29) | <0.00001 | 0 | serious^a^ | not serious | not serious | not serious | publication bias strongly suspected^e^ | Low |
|  | Feng J 2021(38) | RR:0.27(0.13, 0.57) | 0.0005 | 62 | serious^a^ | serious^b^ | not serious | not serious | publication bias strongly suspected^f^ | Very low |
| Electrocardiogram (ECG) efficacy | Zhu XL 2018(21) | OR:2.4(1.69,  3.59) | <0.00001 | 3 | serious^a^ | not serious | not serious | not serious | none | Moderate |
|  | Zhou J 2018(22) | OR:3.2(2.26,  4.54) | <0.00001 | 0 | serious^a^ | not serious | not serious | not serious | none | Moderate |
|  | Zhang HY 2022(24) | OR:3.34(2.44,  4.56) | <0.001 | 0 | serious^a^ | not serious | not serious | not serious | publication bias strongly suspected^f^ | Low |
|  | Wang Y 2012(26) | OR:2.00(1.64, 2.43) | <0.00001 | 0 | serious^a^ | not serious | not serious | not serious | none | Moderate |
|  | Wang KY 2021(27) | RR:1.43(1.20, 1.71) | <0.0001 | 0 | serious^a^ | not serious | not serious | not serious | publication bias strongly suspected^f^ | Low |
|  | Wang HJ 2020(28) | OR:2.35(1.75, 3.16) | <0.00001 | 0 | serious^a^ | not serious | not serious | not serious | none | Moderate |
|  | Tang W 2016(29) | OR:0.52(0.41, 0.66) | <0.001 | 0 | serious^a^ | not serious | not serious | not serious | none | Moderate |
|  | Sun H 2024(30) | MD:1.16(1.04, 1.30) | 0.01 | 0 | serious^a^ | not serious | not serious | serious^c^ | none | Low |
|  | Ren ZX 2018(31) | OR:1.98(1.26, 3.09) | 0.003 | 18 | serious^a^ | not serious | not serious | not serious | publication bias strongly suspected^f^ | Low |
|  | Liu ZC 2020(32) | RR:1.26(1.20, 1.32) | <0.00001 | 0 | serious^a^ | not serious | not serious | not serious | publication bias strongly suspected^f^ | Low |
|  | Liu J 2021(33) | RR:1.29(1.20,  1.38) | <0.00001 | 0 | serious^a^ | not serious | not serious | not serious | none | Moderate |
|  | Li XY 2023(34) | RR:1.32(1.02,  1.71) | 0.03 | 85 | serious^a^ | serious^b^ | not serious | not serious | publication bias strongly suspected^f^ | Very low |
|  | Li L 2022(35) | OR:2.30(1.23,  4.28) | 0.009 | 0 | serious^a^ | not serious | not serious | not serious | publication bias strongly suspected^f^ | Low |
|  | Li CX 2019(36) | OR:1.34(1.26,  1.42) | <0.00001 | 26 | serious^a^ | not serious | not serious | not serious | publication bias strongly suspected^f^ | Low |
|  | Feng J 2021(38) | RR:0.48(0.41, 0.57) | <0.00001 | 47 | serious^a^ | not serious | not serious | not serious | publication bias strongly suspected^f^ | Low |
| Frequency of angina attacks | Zhang XD 2018(23) | RR:1.25(1.16, 1.35) | <0.00001 | 0 | serious^a^ | not serious | not serious | not serious | none | Moderate |
|  | Wang KY 2021(27) | -- | <0.00001 | 97 | serious^a^ | serious^b^ | not serious | not serious | publication bias strongly suspected^f^ | Very low |
|  | Wang HJ 2020(28) | MD:-1.87(-4.06, 0.32) | 0.09 | 98 | serious^a^ | serious^b^ | not serious | serious^c^ | none | Very low |
|  | Sun H 2024(30) | MD:-2.19(-5.04, 0.65) | 0.13 | 100 | serious^a^ | serious^b^ | not serious | serious^c^ | none | Very low |
|  | Liu ZC 2020(32) | SMD:-2.00(-2.78, -1.21) | <0.00001 | 97 | serious^a^ | serious^b^ | not serious | not serious | none | Low |
|  | Liu J 2021(33) | SMD:-2.41(-2.79, -2.02) | <0.0001 | 95 | serious^a^ | serious^b^ | not serious | not serious | none | Low |
|  | Han Y 2022(37) | SMD:-2.26(-4.10, -0.42) | 0.002 | 97 | serious^a^ | serious^b^ | not serious | serious^c^ | publication bias strongly suspected^e^ | Very low |
|  | Du HQ 2020(39) | MD:-2.06(-2.22, -1.91) | 0.00001 | 89 | serious^a^ | serious^b^ | not serious | serious^c^ | publication bias strongly suspected^e^ | Very low |
| Duration of angina attacks | Zhang XD 2018(23) | RR:1.25(1.16, 1.35) | <0.00001 | 0 | serious^a^ | not serious | not serious | not serious | none | Moderate |
|  | Wang KY 2021(27) | -- | <0.00001 | 87 | serious^a^ | serious^b^ | not serious | serious^c^ | publication bias strongly suspected^f^ | Very low |
|  | Sun H 2024(30) | MD:-2.28(-3.44, -1.12) | 0.0001 | 98 | serious^a^ | serious^b^ | not serious | serious^c^ | none | Very low |
|  | Liu ZC 2020(32) | SMD:-2.42(-3.78, -1.06) | 0.0005 | 98 | serious^a^ | serious^b^ | not serious | serious^c^ | none | Very low |
|  | Liu J 2021(33) | SMD:-2.31(-2.65, -1.97) | <0.0001 | 94 | serious^a^ | serious^b^ | not serious | not serious | none | Low |
|  | Du HQ 2020(39) | SMD:-2.03(-3.03, -1.03) | <0.0001 | 93 | serious^a^ | serious^b^ | not serious | serious^c^ | publication bias strongly suspected^e^ | Very low |
| Incidence of cardiovascular events | Wang HJ 2020(28) | OR:0.23(0.07, 0.73) | 0.01 | 0 | serious^a^ | not serious | not serious | serious^d^ | none | Low |
|  | Li XY 2023(34) | RR:0.43(0.30, 0.61) | <0.00001 | 17 | serious^a^ | not serious | not serious | not serious | publication bias strongly suspected^f^ | Low |
| Total cholesterol (TC) | Zhu XL 2018(21) | WMD:0.76(0.23,1.29) | 0.005 | 95 | serious^a^ | serious^b^ | not serious | serious^c^ | none | Very low |
|  | Li L 2022(35) | WMD:-0.37(-0.51,-0.23) | <0.00001 | 0 | serious^a^ | not serious | not serious | serious^c^ | publication bias strongly suspected^f^ | Very low |
|  | Feng J 2021(38) | MD:-0.80(-/094, -0.67) | <0.00001 | 88 | serious^a^ | serious^b^ | not serious | not serious | publication bias strongly suspected^f^ | Very low |
| Triglycerides (TG) | Zhu XL 2018(21) | WMD:0.77(0.20～1.34) | 0.008 | 94 | serious^a^ | serious^b^ | not serious | serious^c^ | none | Very low |
|  | Feng J 2021(38) | MD:-0.44(-0.56, -0.32) | <0.00001 | 92 | serious^a^ | serious^b^ | not serious | not serious | publication bias strongly suspected^f^ | Very low |
| Low-density lipoprotein (LDL) | Zhu XL 2018(21) | WMD:1.85(1.77,1.92) | <0.00001 | 0 | serious^a^ | not serious | not serious | serious^c^ | none | Low |
|  | Li L 2022(35) | WMD:-0.24(-0.43,-0.05) | 0.01 | 0 | serious^a^ | not serious | not serious | serious^c^ | publication bias strongly suspected^f^ | Very low |
|  | Feng J 2021(38) | MD:-0.44(-0.53, -0.35) | <0.00001 | 76 | serious^a^ | serious^b^ | not serious | not serious | publication bias strongly suspected^f^ | Very low |
| High-density lipoprotein cholesterol (HDL-C) | Li L 2022(35) | WMD:0.21(0.04,0.38) | 0.01 | 88 | serious^a^ | serious^b^ | not serious | serious^c^ | publication bias strongly suspected^f^ | Very low |
|  | Feng J 2021(38) | MD:0.13(0.07, 0.19) | <0.0001 | 91 | serious^a^ | serious^b^ | not serious | not serious | publication bias strongly suspected^f^ | Very low |
| High-sensitivity C-reactive protein (hs-CRP) | Zhu XL 2018(21) | WMD:1.23(0.25,2.21) | 0.01 | 93 | serious^a^ | serious^b^ | not serious | serious^c^ | none | Very low |
|  | Zhou J 2018(22) | -- | -- | 96 | serious^a^ | serious^b^ | not serious | not serious | none | Low |
|  | Zhang HY 2022(24) | -- | -- | 93 | serious^a^ | serious^b^ | not serious | not serious | publication bias strongly suspected^f^ | Very low |
|  | Wang HJ 2020(28) | MD:-1.57(-1.73, -1.40) | <0.00001 | 96 | serious^a^ | serious^b^ | not serious | serious^c^ | publication bias strongly suspected^f^ | Very low |
|  | Li XY 2023(34) | MD:-2.75(-3.71, -1.79) | <0.00001 | 93 | serious^a^ | serious^b^ | not serious | serious^c^ | publication bias strongly suspected^f^ | Very low |
|  | Li L 2022(35) | WMD:-2.05(-3.34, -0.76) | 0.002 | 84 | serious^a^ | serious^b^ | not serious | serious^c^ | publication bias strongly suspected^f^ | Very low |
|  | Han Y 2022(37) | MD:-1.28(-1.81, -0.75) | <0.00001 | 55 | serious^a^ | serious^b^ | not serious | not serious | publication bias strongly suspected^e^ | Very low |
| Interleukin-6 (IL-6) | Wang HJ 2020(28) | MD:-16.12(-32.77, 0.53) | 0.06 | 95 | serious^a^ | serious^b^ | not serious | serious^c^ | none | Very low |
|  | Han Y 2022(37) | MD:-4.65(-6.917, -2.39) | <0.0001 | 55 | serious^a^ | serious^b^ | not serious | serious^c^ | publication bias strongly suspected^e^ | Very low |
| Interleukin-18 (IL-18) | Wang HJ 2020(28) | MD:-22.53(-37.02, -8.03) | 0.002 | 78 | serious^a^ | serious^b^ | not serious | serious^c^ | none | Very low |
|  | Han Y 2022(37) | MD:-2.53(-2.84, -2.22) | <0.00001 | 32 | serious^a^ | not serious | not serious | serious^c^ | publication bias strongly suspected^e^ | Very low |
| Whole blood viscosity | Wang HJ 2020(28) | MD:-1.06(-1.30, -0.82) | <0.00001 | 0 | serious^a^ | not serious | not serious | serious^c^ | none | Low |
|  | Feng J 2021(38) | MD:-1.22(-1.69, -0.75) | <0.00001 | 94 | serious^a^ | serious^b^ | not serious | not serious | publication bias strongly suspected^f^ | Very low |
| Plasma viscosity (PV) | Yang T 2022(25) | MD:-0.46(-0.59,-0.33) | <0.00001 | 97 | serious^a^ | serious^b^ | not serious | not serious | none | Low |
|  | Wang HJ 2020(28) | MD:-1.06(-1.30, -0.82) | <0.00001 | 0 | serious^a^ | not serious | not serious | serious^c^ | none | Low |
|  | Feng J 2021(38) | MD:-0.31(-0.40, -0.21) | <0.00001 | 78 | serious^a^ | serious^b^ | not serious | not serious | publication bias strongly suspected^f^ | Very low |
| Hematocrit | Wang HJ 2020(28) | MD:-2.06(-4.38, -0.82) | 0. 004 | 85 | serious^a^ | serious^b^ | not serious | serious^c^ | none | Very low |
| Fibrinogen | Wang HJ 2020(28) | MD:-0.73(-1.04, -0.41) | <0.00001 | 0 | serious^a^ | not serious | not serious | serious^c^ | none | Low |
| Endothelin-1 (ET-1) | Li XY 2023(34) | MD:-9.34(-11.36, -7.32) | <0.00001 | 20 | serious^a^ | not serious | not serious | not serious | publication bias strongly suspected^f^ | Low |
|  | Li L 2022(35) | WMD:-12.71(-15.00, -10.42) | <0.00001 | 0 | serious^a^ | not serious | not serious | serious^c^ | publication bias strongly suspected^f^ | Very low |
| Endothelial function marker nitric oxide (NO) | Zhang HY 2022(24) | SMD:1.29(1.06,1.52) | <0.001 | 0 | serious^a^ | not serious | not serious | serious^c^ | publication bias strongly suspected^f^ | Very low |
|  | Han Y 2022(37) | MD:8.03(0.93, 15.14) | 0.03 | 96 | serious^a^ | not serious | not serious | serious^c^ | publication bias strongly suspected^e^ | Very low |
| Left ventricular ejection fraction (LVEF) | Zhou J 2018(22) | MD:4.93(3.39, 6.47) | <0.00001 | 0 | serious^a^ | not serious | not serious | serious^c^ | none | Low |
|  | Zhang HY 2022(24) | -- | -- | 93 | serious^a^ | serious^b^ | not serious | not serious | publication bias strongly suspected^f^ | Very low |
| N-terminal pro b-type natriuretic peptide (NT-proBNP) | Zhang HY 2022(24) | SMD:-270.49(-298.44,-242.34) | <0.001 | 0 | serious^a^ | not serious | not serious | serious^c^ | publication bias strongly suspected^f^ | Very low |
| Myeloperoxidase (MPO) | Yang T 2022(25) | MD:-6.25(-7.26,-5.24) | <0.00001 | 90 | serious^a^ | serious^b^ | serious^c^ | not serious | none | Very low |
| Dosage of nitroglycerin | Wang KY 2021(27) | MD:-3.81(-5.45,-2.17) | <0.00001 | -- | serious^a^ | serious^b^ | not serious | serious^c^ | publication bias strongly suspected^f^ | Very low |
| Duration of exercise | Li XY 2023(34) | MD:59.33(39.16,79.90) | <0.00001 | 72 | serious^a^ | serious^b^ | not serious | not serious | publication bias strongly suspected^f^ | Very low |

**Explanations:**

a The RCTs included in the had poor methodological quality, inducing a risk of bias.

b The heterogeneity test obtained I^2^＞50%, P<0.01.

c The sample size of continuous variables was <400.

d The sample size of binary variables was <300.

e A small sample size with positive results.

f. Low methodological quality.
